# A Network-Based Classification Model for Deriving Novel Drug-Disease Associations and Assessing Their Molecular Actions

**DOI:** 10.1371/journal.pone.0111668

**Published:** 2014-10-30

**Authors:** Min Oh, Jaegyoon Ahn, Youngmi Yoon

**Affiliations:** 1 Department of Computer Engineering, Gachon University, Seongnam, Korea; 2 Department of Integrative Biology and Physiology, University of California Los Angeles, Los Angeles, California, United States of America; Cincinnati Childrens Hospital Medical Center, United States of America

## Abstract

The growing number and variety of genetic network datasets increases the feasibility of understanding how drugs and diseases are associated at the molecular level. Properly selected features of the network representations of existing drug-disease associations can be used to infer novel indications of existing drugs. To find new drug-disease associations, we generated an integrative genetic network using combinations of interactions, including protein-protein interactions and gene regulatory network datasets. Within this network, network adjacencies of drug-drug and disease-disease were quantified using a scored path between target sets of them. Furthermore, the common topological module of drugs or diseases was extracted, and thereby the distance between topological drug-module and disease (or disease-module and drug) was quantified. These quantified scores were used as features for the prediction of novel drug-disease associations. Our classifiers using Random Forest, Multilayer Perceptron and C4.5 showed a high specificity and sensitivity (AUC score of 0.855, 0.828 and 0.797 respectively) in predicting novel drug indications, and displayed a better performance than other methods with limited drug and disease properties. Our predictions and current clinical trials overlap significantly across the different phases of drug development. We also identified and visualized the topological modules of predicted drug indications for certain types of cancers, and for Alzheimer’s disease. Within the network, those modules show potential pathways that illustrate the mechanisms of new drug indications, including propranolol as a potential anticancer agent and telmisartan as treatment for Alzheimer’s disease.

## Introduction

Drugs cure diseases by targeting the proteins related to the phenotypes arising from the disease. However, drug development does not precisely follow the “one gene, one drug, one disease” paradigm, which has been challenged in many cases. The concept of polypharmacology was proposed for drugs acting on multiple targets rather than one target [1], [2]. The polypharmacological concept can lead to drug repositioning, which involves finding new indications for existing drugs or side effects due to the molecular mechanisms that may underlie a chemical–disease connection [3],[4].

In order to decipher how drugs exert their effect on diseases, it is important to understand how a drug acts on targets related to a disease phenotype, how a gene module causes an abnormal phenotype, and how, in consequence, the targets and causative genes interact with each other. Furthermore, it is of great importance to investigate how drugs exert their activities directly or indirectly via such gene modules, how patho-phenotypes are influenced by the abnormality of gene modules, and how drugs and disease phenotypes are associated on the basis of gene modules [5]. With this understanding, identifying and analyzing how a drug and a disease are actually associated at the molecular level plays a crucial role in the prediction of new drug indications.

Currently, computational methods to predict potential drug-disease interactions can be divided into the drug-centric approach, the disease-centric approach, and the drug-disease mutual approach.

With the drug-centric approach, opportunities are sought to repurpose drugs using accumulated chemical or pharmaceutical knowledge. Keiser et al. applied an integrated chemical similarity approach to drug repositioning using structural similarities among drug compounds and knowledge of established compound-target relationships [6]. However, many physiological effects cannot be predicted by chemical properties alone because drugs undergo complex, largely uncharacterized metabolic transformations as they are metabolized and physiologically distributed [7].

The disease-centric approach mainly utilizes the characteristics of diseases from the perspective of disease management, symptomatology, or pathology [7]. This approach builds a diseasome, or group of diseases, by incorporating established knowledge about diseases, or it finds and uses the common characteristics of diseases associated with an existing drug. Hu and Agarwal established a disease-similarity network using gene expression profiles and incorporated into this network a body of knowledge about drugs [8]. Suthram et al. constructed a disease network and discovered functional modules common to diseases that are enriched for pluripotent drug target genes [9]. This disease-only-based approach relies heavily on data denoting the characteristics of diseases, and it can be affected by the quality of the data. Therefore, outcomes could be restricted according to the means used to measure gene expression profiles or phenotypic profiles, which represent the characteristics of diseases.

The drug-disease mutual approach is a combination of the two approaches described above. It can infer new therapeutic relationships between drugs and diseases by directly matching the biomolecular or chemical properties of drugs, or processed data pertaining to these properties, with the property data or processed data of diseases. Alternatively, it can infer relationships indirectly using related or higher-level data or representations of drugs and diseases. Utilizing knowledge of both drugs and diseases can be a complementary and successful strategy; in particular, this approach can overcome missing knowledge with regard to the pharmacology of a drug, such as unknown or additional targets [7].

Among drug-disease mutual approaches, one study that directly matched the properties of drugs and diseases constructed a signature of a drug and a signature of a disease using gene expression microarrays. This approach identified new therapeutic potentials of the drug by matching the two signatures [10]. Another attempt introduced the concept of a co-module, which is a representation of a drug-gene-disease relationship [11]. A network-based gene-closeness profile was defined to relate the drug to the disease, and new drug-disease associations were identified.

As another drug-disease mutual approach, Gottlieb et al. indirectly utilized the properties of drugs and diseases [12]. Based on the observation that similar drugs are indicated for similar diseases, they constructed drug-drug and disease-disease similarity measures and exploited these measures to construct classification features, with the subsequent learning of a classification rule. For reproducible implementation, it is limited to gather all the required properties of drugs and diseases.

With the increasing number and variety of high-throughput datasets, functional genetic networks are becoming more accurate and complete. These networks make it possible to understand how drugs and diseases are associated at the molecular level. If the network features of drug-disease associations can be properly selected, these can be used to infer novel indications or side effects of existing drugs with increased accuracy, providing more concrete evidence.

Here, we propose scoring methods to quantify drug-disease relationship. Network adjacencies of drug-drug, disease-disease were quantified. Furthermore distance between topological module of drug-drug and disease, and distance between topological module of disease-disease and drug were quantified. These quantified scores were used as features for the prediction of novel drug-disease associations. Our method obtains an AUC of 0.845 when predicting drug-disease associations and shows better performance compared to other methods. We confirmed that our prediction method covers a number of current clinical trials (over 34%). Also, we extracted the topological modules of novel predictions, which involve propranolol for certain types of cancer and telmisartan for Alzheimer’s disease. The module of propranolol shows significant enrichment in cancer pathways and putative inhibition mechanism of cancer growth and proliferation. In addition, the module of telmisartan indicates its therapeutic action related with inhibition of the defective signaling that usually occurred in Alzheimer’s disease. Our approach provides promising drug-disease relationships for drug repositioning and reveals potential mechanism of them.

## Methods

**Figure 1:**
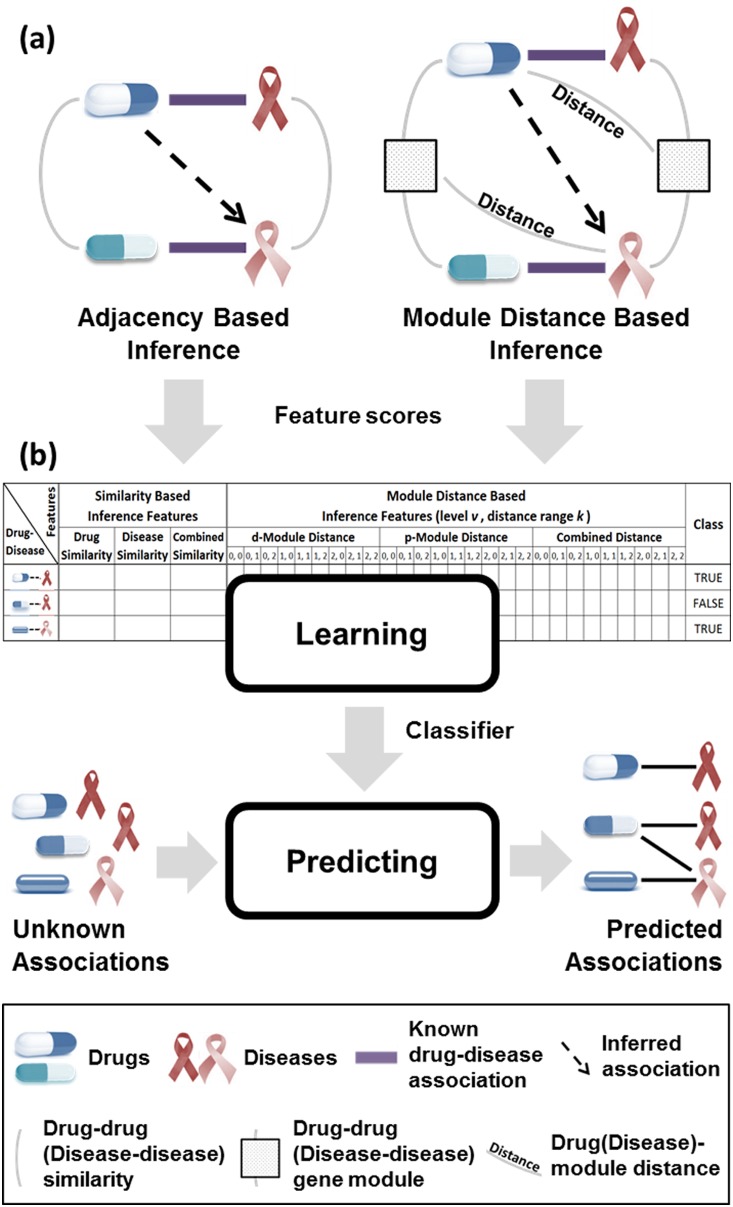
System overview. (a) “Adjacency-Based Inference” measures the drug-drug (disease-disease) adjacency among known drug-disease associations, and infers new drug-disease association. “Module-Distance-Based Inference” derives drug-drug (disease-disease) gene module among known drug-disease associations, measures the distance between the gene module and disease (drug), and infers new drug-disease association. (b) Drug-disease relationship represented by score becomes features. Various machine learning based classifiers are built with those features, and predict unknown drug-disease relationship.

The proposed method consists of two processes, as shown in Figure 1. In the first stage, the degree of the drug-disease association is scored by means of adjacency-based inference and module-distance-based inference. Detailed descriptions of the adjacency-based inference and module-distance-based inference methods are given in section “Two methods for scoring drug-disease associations”. In the second stage, the scores from the first stage are regarded as features characterizing the drug-disease relationship; a classifier is subsequently built using these features by means of learning. With this classifier, predictions are made regarding whether an unknown drug-disease pair has an association. Finally, new drug-disease associations are discovered. The details of this stage are given in section “Characterizing a drug-disease relationship via features”.

### Datasets

We used an integrative genetic network that combines three types of protein and gene networks. They comprised 152,388 protein-protein interactions from the Online Predicted Human Interaction Database (version 2.0) [13], 13,106 gene regulation data consisting of 13,046 activation and 1,085 inhibition interactions, and 16,302 inferred protein-protein interactions from known protein complexes in the human pathways of the Pathway Interaction Database [14]. We assumed that each protein in a protein complex also has an interaction that is equal to the interactions of the protein complex. For instance, we can get four binary interactions when a protein complex with four proteins has an interaction with a gene [44]. In total, we used 177,672 interactions for the integrative genetic network, with 15,804 unique proteins. To integrate the networks, we mapped the proteins and genes with UniProt ID.

We obtained drugs and their targets from DrugBank [15]. We selected drugs FDA approved as candidate drugs having multiple targets (≥2) on the integrative genetic network, resulting in 832 drugs and 4,889 drug-target relationships. Diseases and their susceptible genes were sourced from OMIM (Online Mendelian Inheritance in Man) [16]. We secured 239 diseases having multiple susceptible genes (≥2) in the integrative network and 4,013 disease-gene relationships. We note that, since drug targets and disease susceptibility genes are not completely revealed and the networks including PPI and the gene regulatory network are incomplete, we used drugs and diseases with multiple target genes and susceptibility genes.

From the Comparative Toxicogenomics Database (CTD), we obtained known drug-disease associations (Sep. 2013, downloaded) [17]. The CTD consists of two types of chemical (drug)-disease relations: curated and inferred. We used only curated relations. We mapped chemicals to their DrugBank identifiers and diseases to their OMIM identifiers. Through the mapping processing, we obtained 5,201 drug-disease associations. We selected drug-disease instances with identifiers belonging to the list of drugs and diseases in our set. Finally, 1,295 known associations consisting of 377 drugs and 80 diseases were used in this study.

### Two methods for scoring drug-disease associations

#### 1) Adjacency-Based Inference

We devised a scoring method for drug-disease relationships based on known drug-disease associations and adjacencies. In this method, three approaches are used: drug-adjacency-based inference, disease-adjacency-based inference, and combined adjacency inference.

**Figure 2:**
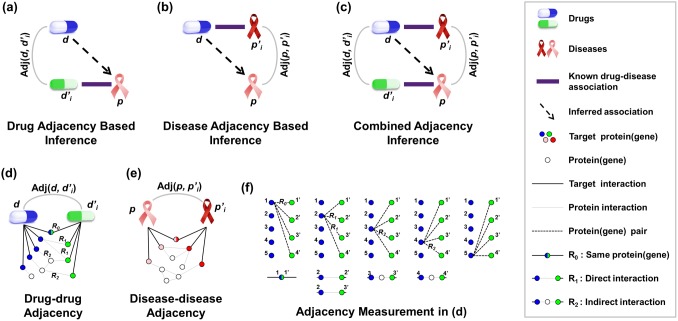
Adjacency-Based Inference.

The basic idea of drug-adjacency-based inference stems from the hypothesis that if there is a known association between a drug and a disease, another similar drug would also have an association with the disease. Figure 2 (a) describes this concept. When *d’* is the drug and *p* is the disease in a known association, *d*, which is adjacent to *d’*, can be inferred to have an association with *p*. It can be said that the inferred association is stronger if the adjacency score is higher; therefore, the drug-drug adjacency score of *d* and *d’* is used as the measure of the inferred association.

The disease-adjacency-based inference approach infers a drug-disease association based on disease-disease adjacency. It is described in Figure 2 (b).

The combined-adjacency inference approach combines the scores from drug-adjacency-based inference and disease-adjacency-based inference. Although the ways in which drug-target proteins and disease-genes work during the biological process differ, the same scoring method is applied. This method prevents one of the scores from being given a greater weight and considers both compounds’ mechanisms of action and disease molecular pathologies.

These three approaches are heavily affected by the degree of adjacencies of the drug-drug or disease-disease relations. A higher adjacency score implies a tighter drug-disease association. Measuring the adjacency score is crucial. We used target proteins for drugs and disease genes for diseases. In the integrative genetic network, the closeness of the association between each target protein set of two drugs is measured, and this measure is used as the drug-drug adjacency score. Also, the closeness of disease gene sets in the integrative network is used for the disease-disease adjacency score. Figures 2 (d) and (e) display examples of drug-drug (disease-disease) adjacency.

Figure 2 (f) displays the process of scoring the drug-drug adjacency in Figure 2 (d). In the integrative genetic network, when finding a shortest path for each pair of proteins, one protein from drug *d* and another protein from drug *d’* are shown. There are three types of paths: R0, R1, and R2. In R0, the target protein of *d* and the target protein of *d’* are identical. In R1, the target protein of *d* and the target protein of *d’* are directly connected. In R2, the target protein of *d* and target protein of *d’* are indirectly connected by means of another protein between them. If the length of a path is shorter, the score is higher. The scores of all pairs are summed and then scaled according to the number of targets. The scaled score becomes the drug-drug adjacency score, as follows:
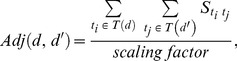
(1)


Here, *t_i_* is a target protein of drug *d* in its target set *T(d)*, and *t_j_* is a target protein of drug *d’*; *scaling factor* essentially denotes the number of proteins in *T(d)* multiplied by the number of proteins in *T(d’),* which allows 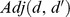 to vary from 0 to 1. Additionally, 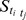
is the shortest path between *t_i_* and *t_j_*, and it is one of three types of paths, i.e., *R0*, *R1*, and *R2*. We set the score of *R0* to the reciprocal number of median degree of a network, which is one sixth. We then set *R1* and *R2* to the square of *R0* and the cube of *R0*, respectively. Among the drug-drug adjacency scores for multiple drugs, the maximum value becomes the final score for the association between *d* and *p*, as follows:
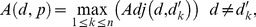
(2)


In this equation, *n* is the number of drugs that have a known association with disease *p*. We computed disease-disease adjacency scores in a similar manner using disease genes in the network. The scores for the association between *d* and *p* from the drug-drug and disease-disease adjacency scores are combined into a single score by computing their weighted geometric mean, as follows:
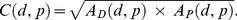
(3)Each 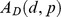 and 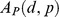 value indicates each maximum drug-drug and disease-disease adjacency score for the association between *d* and *p*.

#### 2) Module-Distance-Based Inference

We selected a topologically related gene set that is called a topological module. The topological module that is shared by two drugs is extracted for a particular disease. One drug is from a known drug-disease association and the other is among the candidate drugs for the disease. This topological module common to two drugs is called d-module, and is used for repurposing a new drug-disease association. In the same manner, a topological module that is common to two diseases is called p-module, and is extracted to discover new drug-disease associations. We applied three approaches: d-module distance-based inference, p-module distance-based inference, and a combined module-distance inference method.

**Figure 3:**
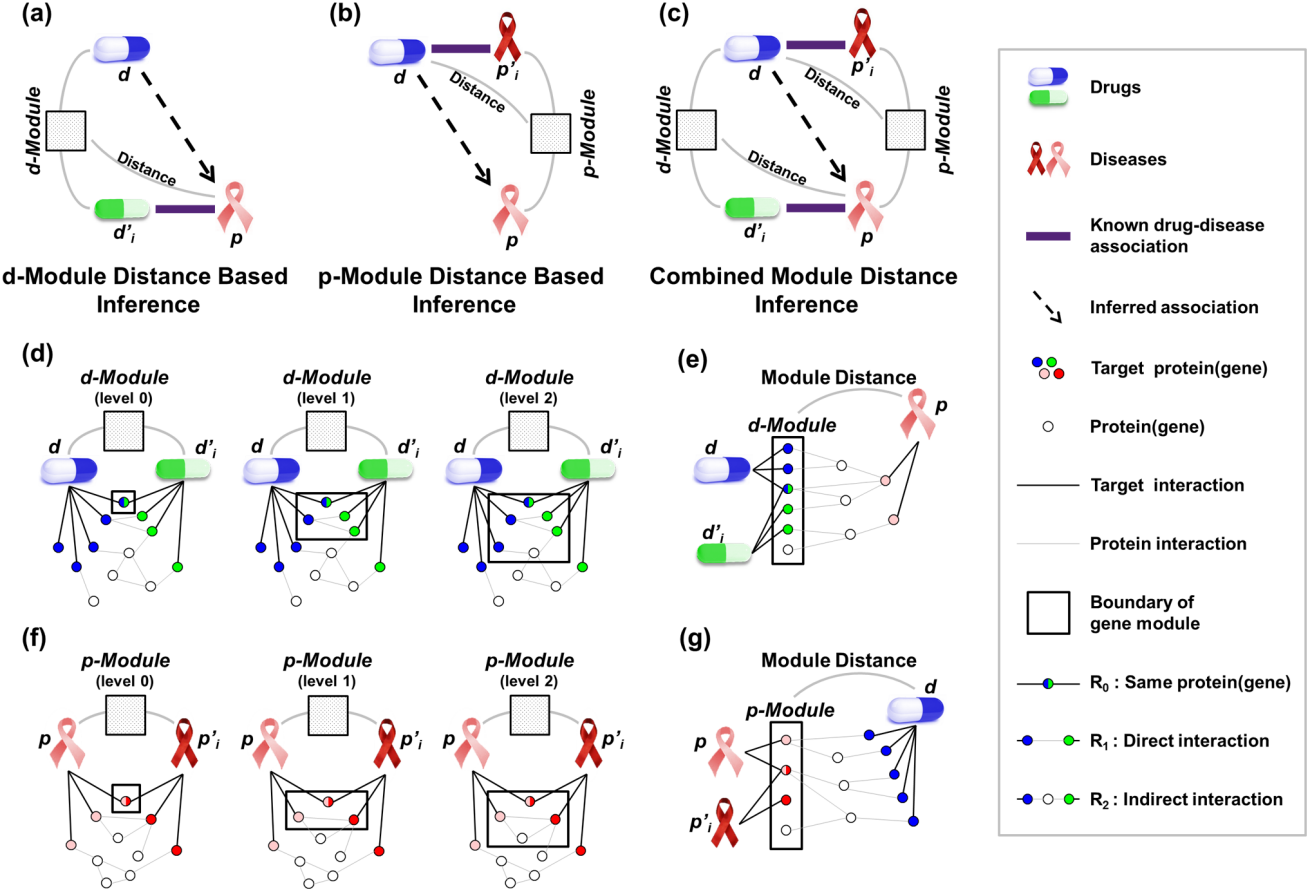
Module-Distance-Based Inference.

From the two drugs (*d*, *d’*), the common topological drug-drug gene module (d-module) is derived and the distance between the d-module and the disease is measured, as described in Figure 3 (a). This module distance indicates how closely common features of the drugs actually relate to the disease. In our assumption, the higher the distance value, the more likely it is that the pathway of the drug in the known drug-disease association is shared with the other drug’s expected pathway.

Inferring drug-disease association by means of the drug-drug topological module is named as d-module distance-based inference. As shown in Figure 3 (d), every interaction in each target protein set from *d* and *d’* is displayed in the integrative genetic network, and the d-module is extracted according to the level parameter *v*. The distance between the d-module at each level and the disease is measured according to the distance parameter *k*. In Figure 3 (e), the module distance between the d-module in level 2 and the disease is measured. Given drugs *d* and *d’* and disease *p*, the d-module distance is computed as follows:
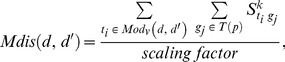
(4)


Here, *t_i_* is a protein of d-module 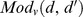 and *g_j_* is a gene of disease *p*; *scaling factor* essentially denotes the number of proteins in 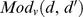 multiplied by the number of genes in *T(p),* which allows 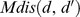 to vary from 0 to 1. Also, 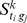 is the score of the path between *t_i_* and *g_j_*; *k* is a fixed length of the path, which is used to calculate 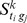. The value of 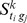 changes according to *k*, as follows:
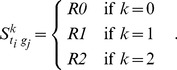
(5)


In the case of *k* = 0, only the intersections between *t_i_* and *g_j_* receive a score. When *k* = 1, only the paths whose length is one receive a score. When *k* = 2, only the paths whose length is two are scored. We set the score of *R0* to the inverse number of median degree of a network, which is one sixth. We then set *R1* and *R2* to the square of *R0* and the cube of *R0*, respectively. Among the module distances for multiple drugs, the maximum values become the final score for the association between *d* and *p*, as follows:
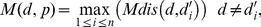
(6)In this equation, *n* denotes the number of drugs with a known association with disease *p*. When the d-module of *d* and *d’* is closely related to the disease genes, we can expect that the two drugs show a similar biological function.

In the p-module distance-based inference method, the common topological disease-disease gene module (p-module) from two diseases is extracted, as shown in Figures 3 (b), (f), and (g). The p-module represents the pathological molecules shared by the two diseases. The shorter the module distance between the p-module and the drug, the more safely it can be assumed that the drug also works for the other disease. The method of calculating the distance follows the d-module-based inference method.

The combined module-distance inference method is a combination of the previously described d-module distance-based inference and the p-module distance-based inference methods. It is expressed as follows:
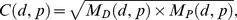
(7)Here, 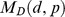 and 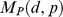 indicate the maximum of the d-module distance-based inference and p-module distance-based inference methods, respectively. It makes the two methods complementary while considering not only the molecular activity shared by the drugs but also the causative genes shared by the diseases (Figure 3 (c)).

### Characterizing a drug-disease relationship via features

The scores from adjacency-based inference and module-distance-based inference are converted into features, and a classifier predicting a new drug-disease association is thereby learned. The drug-adjacency-based inference score, disease-adjacency-based inference score, and combined adjacency inference score from the adjacency-based inference method become the first three features for each drug-disease relationship.

The d-module distance-based inference scores, p-module distance-based inference scores, and combined module-distance inference scores from the module-distance-based inference are used as 27 features. In the process of calculating the module, we set the level parameter *v* to range from 0 to 2, which denotes the scope of the module. When calculating the shortest path between proteins in a module and the target proteins (disease genes) of a drug (disease), we set the distance parameter *k* to range from 0 to 2, which determines the shortest paths (R0∼R2 in Figure 3). Accordingly, 27 features are generated from the module-distance-based inference method. Each drug-disease pair has 30 features overall, including 3 features from the adjacency-based inference. We note that calculating all 30 features for 2,590 positive and negative associations takes about five minutes on Intel Core i7 CPU (3.50 GHz).

The training set used for 10-fold cross-validation includes 1,295 known drug-disease associations as a positive set and randomly generated drug-disease pairs as a negative set. The negative set is randomly generated from drugs and diseases in the positive set, taking the same size as the positive set. We note that a random negative set might give optimistic results, but it is challenging to create an exact negative set. To obtain a precise AUC score, 10-fold cross-validation is independently conducted 10 times. Each cross-validation is conducted with different random negative sets, and generates an AUC score. We then average the resulting AUC scores. Table S1 shows one sample of an actual training data set. A classifier is learned with these features. Ten-fold cross-validation is done for a performance evaluation.

## Results

We selected 1,295 known drug-disease associations from the CTD and their elements, along with 377 drugs and 80 diseases. The integrative genetic network used here consists of a gene regulation database and inferred and experimental protein interaction databases. For each drug-disease pair, specific feature scores are calculated using adjacency-based inference and module-distance-based inference on top of the integrative genetic network, and a classifier is learned with the feature scores. This classifier predicts unknown drug-disease associations.

### Performance evaluation

**Figure 4:**
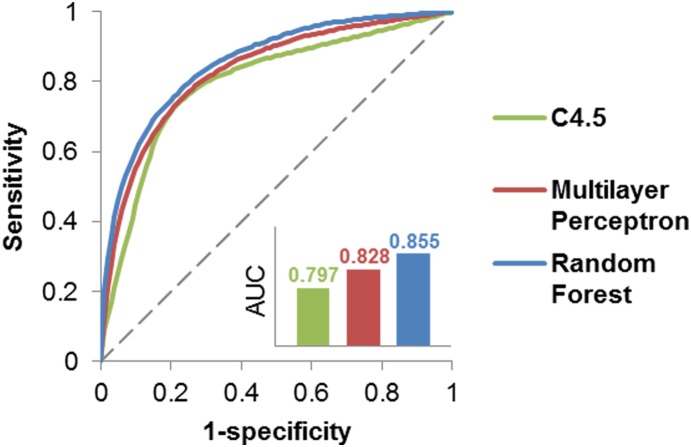
Ten-fold cross-validation.

For performance testing, independent 10-fold cross-validations were conducted ten times. The training set in each 10-fold cross-validation consisted of a positive set of true drug-disease associations and a negative set of randomly generated drug-disease pairs. Each training set was arbitrarily separated into 10 parts (trained on nine of them and tested on the remaining one), and the process was repeated ten times for cross-validation. This procedure was applied to all of the ten different training sets, and a random negative set was respectively generated for each of them. The resulting AUC scores of the ten 10-fold cross-validations were averaged. We used C4.5, Multilayer Perceptron and Random Forest, as implemented in Weka v3.6 [18]. The 10-fold cross-validation results with the 10 training data sets are shown in Figure 4 and Table S2. The highest AUC (area under the ROC) is 0.855.

**Figure 5:**
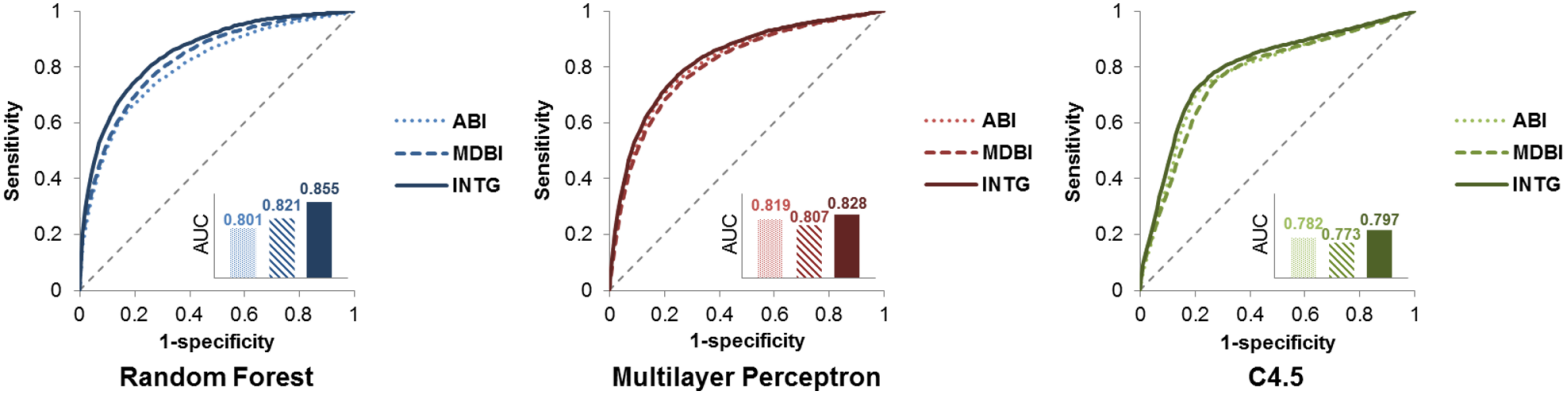
Performance evaluation of each method. Results from the adjacency-based inference (ABI) method, the module-distance-based inference (MDBI) method, and the integrated method of ABI and MDBI (INTG) are compared.

**Figure 6:**
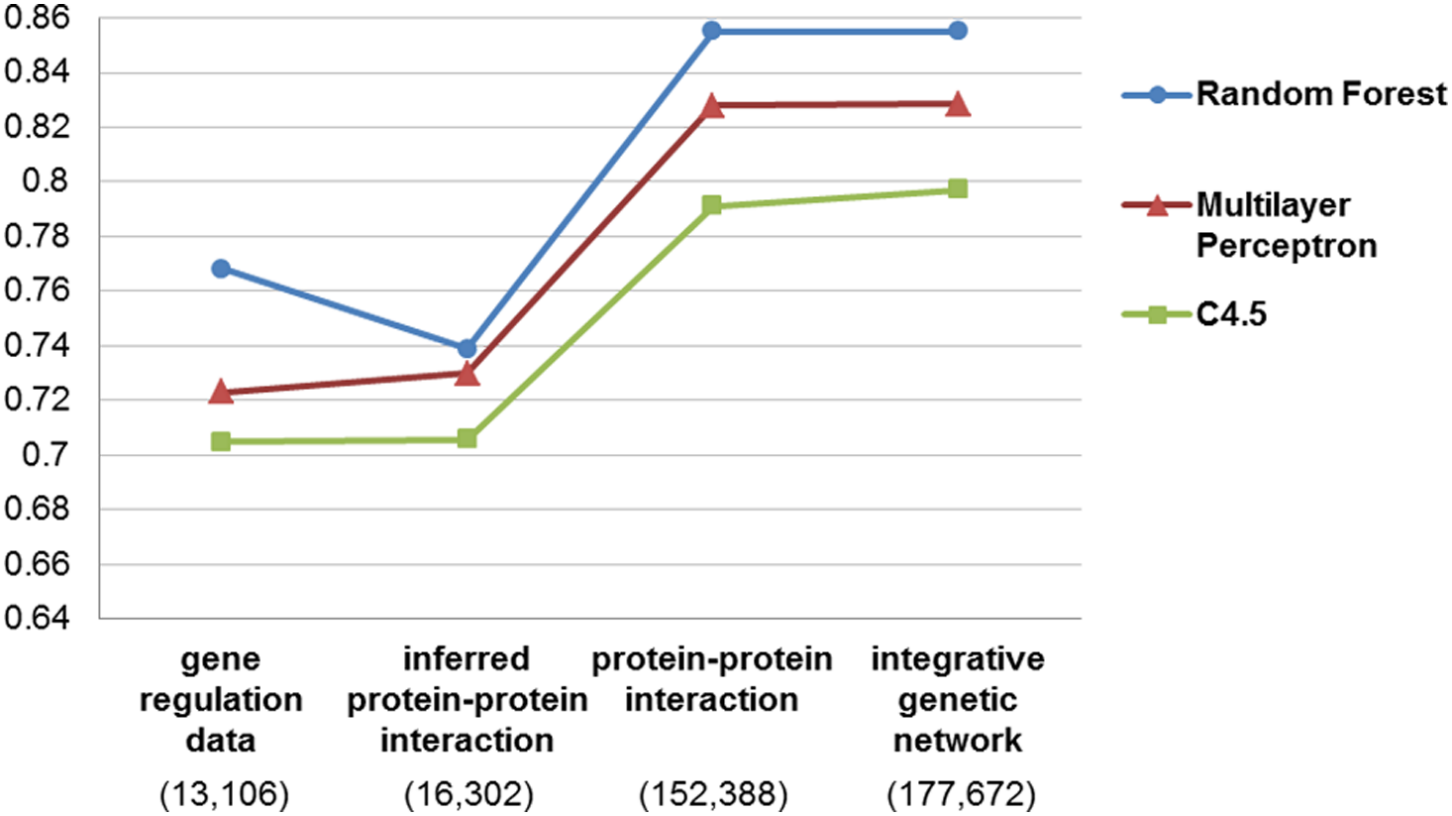
AUC comparison of three individual networks and the integrated network.

In our study, two methods were implemented for scoring the features. Figure 5 shows the AUC when each method was used alone and when both methods were used. The performance when both methods were used exceeds that when only one method was used. In Table S3, we evaluated the contribution of all features, when both methods were used. We also used the integrative genetic network, which consists of gene and protein interaction databases. Figure 6 shows the AUC of three individual networks and the integrative network. Using PPI only results in a higher AUC compared to the use of gene regulation data alone or the use of the inferred PPI alone. Additionally, using integrative networks shows a slightly better AUC for C4.5 compared to the use of PPI alone.

### Comparison with other methods

We compared our method with two previous methods, PREDICT [12] and CMap [19]. PREDICT observes similar drugs that are indicated for similar diseases based on multiple drug-drug and disease-disease similarity measurements. PREDICT uses a gold standard set of drug-disease associations as known associations. We obtained our classification result using this gold standard set and compared their classification performances. Out of 1,933 associations, 247 known associations, composed of 179 drugs and 80 diseases that have multiple targets and multiple susceptibility genes, were used to make the training set. The AUC score of our method shows slight better performance (AUC = 0.917) than that of PREDICT (AUC = 0.900).

CMap searches for drug response gene-expression profiles that relate to the disease signature and predicts drug-disease associations. We downloaded 21 disease gene signatures out of 80 diseases in our study from ArrayExpress [42]. Given that CMap is restricted to include only signatures having up-regulated or down-regulated genes and to include signatures not exceeding 1,000 genes, we were able to secure only five disease signatures. The number of drugs used in both our method and CMap is 201. There are 31 known drug-disease associations between 201 drugs and five diseases. These 31 known associations and their elements, 30 drugs and four diseases, were used for a comparison. Even with this limited number of known drug-disease associations, the AUC in our method is 0.991, while the AUC of CMap is 0.360. In the , we show AUC reports of CMap based on the different proportion of input genes.

### New predictions

Among 30,160 drug-disease pairs (377 drugs and 80 diseases), we predicted 6,143 novel drug-disease associations using the classifier (Table S4). We compared our predictions and clinical trials for validation, and 7,854 unique drug-disease associations were obtained from a registry of publicly and privately conducted worldwide clinical studies (http://clinicaltrials.gov/). The MeSh term was used to map the conditions of the clinical trials to an MIM number and to map interventions to DrugBank entries. Out of 7,854 associations, 942 associations involve drugs and diseases that are present in our data set. The coverage rate of the 6,143 predicted associations with respect to 942 clinical trial associations is 36.2 percent as shown in Table 1 (Fisher’s exact P = 4.02E-30). In order to validate that the prediction is not trivial due to structurally same drugs, we computed chemical similarity between known drugs and predicted drugs for same disease. We calculated Tanimoto score between drugs based on their fingerprints downloaded from DrugBank. Among all the drug pairs, only 0.58% of them show chemical similarity (Tanimoto coefficient >0.7) as displayed in Figure S1.

**Table 1:** Statistics of the Predictions in the Clinical Trials.

	377 drugs and 80 diseases				832 drugs and 239 diseases			
Phases	Associations inclinical trials	Associations inprediction	Coverage	p-value	Associations inclinical trials	Associations inprediction	Coverage	p-value
Total	942	341	36.2%	4.02E-30	1100	405	36.8%	3.71E-84
Phase 1	243	79	32.5%	3.13E-24	287	93	32.4%	2.11E-07
Phase 1/2	124	37	29.8%	4.61E-50	150	41	27.3%	5.17E-28
Phase 2	466	159	34.1%	0.006636	532	180	33.8%	0.006982
Pahse 2/3	60	15	25.0%	2.23E-71	66	17	25.8%	1.01E-46
Phase 3	249	57	22.9%	5.40E-36	297	73	24.6%	3.94E-13
Phase 4	214	55	25.7%	3.25E-37	244	75	30.7%	1.53E-12
Unlisted	251	77	30.7%	3.46E-25	296	95	32.1%	5.51E-07

Total numbers are unique associations, excluding redundancy between phases.

We also examined extended drug-disease pairs that consist of 832 FDA-approved drugs extracted from DrugBank and 239 diseases taken from OMIM. Among 198,848 drug-disease pairs, 26,909 associations were predicted using our classifier (Table S5). Out of 7,854 clinical trials, 1,100 associations involve drugs and diseases that are present in the extended data set. The coverage rate of the predicted associations with respect to 1,100 clinical trial associations is 36.8 percent (Fisher’s exact P = 3.71E-84). These two coverage rates are relatively high compared to that reported by Gottlieb et al., who demonstrated coverage of 27 percent [12].

## Discussion

### Path types used in the genetic network

To measure the network traits between genes derived from drug and disease, we defined three types of paths between two genes: R0, R1 and R2. First, R0 denotes that the two genes are identical. Second, R1 indicates that the two genes are linked by direct interaction. Third, R2 means that indirect interaction through another gene connects two genes (see method section). We conduct the independent experiments using only single type of gene pair and comparing them with original method which uses all types of gene pairs. Table S6 shows that it is better to consider all the paths together than consider only single type of path in improving classification performance. Additionally, in most cases, the classifier using only R2 shows higher performance than the classifier using only R0 or R1. Table S3 indicates the feature contribution of these paths by changing *k* value, which means alteration of path type. The features of which k = 2, meaning only R2 being used, generally show higher rank than other features where k = 1 or k = 0, and the features of which k = 1 usually have higher information gain than other features where k = 0.

### Beta-adrenergic antagonist as a potential cancer treatment

First, we focused on a potential therapeutic agent as a cancer treatment. Out of 26,909 new predictions, 5,809 cancer-specific associations were selected according to the disease category from earlier work [20]. Nearly 5 percent of the drugs in these associations target beta-adrenergic receptors. Several recent epidemiological studies have shown that the use of beta-blockers reduces the progression and secondary formation of cancer and improves the potential for relapse-free survival in patients with cancer [21]–[24]. Also, Al-wadei et al. indicated that the inhibition of beta-adrenergic signaling can lead to potential anti-cancer drug development [25]. Our 162 cancer-related predicted associations include 21 drugs out of the 24 approved beta-adrenergic blockers from DrugBank. Generally, beta-adrenergic blockers are known as antihypertensive agents, and their blood pressure regulation pathways are well known. However, a few studies recently explained the mechanism of pathways related to the initiation and progression of cancer.

**Figure 7:**
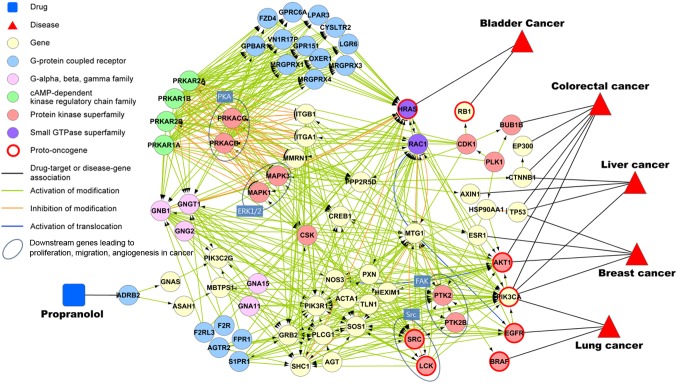
Gene regulatory network between ADRB2 and cancer-specific genes.

To elucidate the role of beta-blockers as a cancer treatment, we displayed a predicted gene network extending from propranolol to cancers (Figure 7). The gene regulatory network and disease-gene are used to construct the network. Propranolol is an antihypertensive agent that is also a beta-adrenergic antagonist. Several cancers are predicted to have an association with propranolol in our study. Propranolol inhibits beta-adrenergic signaling, as shown in Figure 7. It is known that the stimulated beta-adrenergic receptor activates Gα_s_ guanine nucleotide-binding protein, resulting in the activation of adenylyl cyclase and the subsequent formation of cyclic adenosine 3′,5′-monophosphate (cAMP) [26]. In addition, the cAMP activation of protein kinase A (PKA) phosphorylates cyclic AMP-responsive element-binding protein (CREB) and transactivates epidermal growth factor receptor (EGFR) [27]. Also, cAMP activates serine/threonine-protein kinase B-raf (BRAF) and mitogen-activated protein kinase (MAPK), leading to the PKA-dependent activation of downstream kinases such as Src kinase (Src) and focal adhesion kinase (FAK) [28]. These kinases and proteins are proto-oncogenes or play a role in the network as an activator of proto-oncogenes, as shown in Figure 7. The gene set in the network was significantly enriched in the cancer pathways such as *prostate cancer* (P = 1.24E-28, FDR = 5.64E-27), *pathways in cancer* (P = 1.11E-27, FDR = 2.53E-26), and *glioma* (P = 2.4E-26, FDR = 4.37E-25), using KEGG biological pathways.

Figure 7 shows that several genes, including HRAS, RAC1, AKT1, and PIK3CA, which usually undergo somatic mutations in specific cancers, play an important role in the pathway. In Figure 7, the cAMP-dependent kinase regulatory chain family and PKA activate GPCRs to cause the activation of HRAS. Also, RAC1, AKT1, and PIK3CA are stimulated by various genes, including FAK, Src, G-beta, the gamma family, and the gene group in the middle of the network. When PIK3CA and AKT1 are downstream effectors of RAS, both HRAS and RAC1 are RAS superfamily of small GTPases that cause cancer growth, invasion, and metastasis [29]. In summary, our network explains the major mechanism of the beta-adrenergic signaling pathway that is related to the RAS superfamily and its downstream genes that cause specific cancers.

### Elucidating the mechanism of telmisartan with regard to Alzheimer’s disease

Many therapeutic agents for Alzheimer’s disease aim to achieve symptomatic benefits; however, currently no disease-modifying therapies are approved. Drug repositioning for Alzheimer’s disease is being considered as an efficient strategy, with several classes of repositioning drugs presented in recent studies. Among the potential repositioning drugs, peroxisome proliferator-activated receptor-γ (PPARγ) agonists and angiotensin receptor blockers have been spotlighted for Alzheimer’s disease [30].

Telmisartan is a therapeutic agent that is prescribed for hypertension. It is known as a unique angiotensin II receptor blocker with PPARγ agonistic properties, and it was predicted to be associated with Alzheimer’s disease in our study. Amyloid-β (Aβ) deposition is a key pathological hallmark of Alzheimer’s disease, and it was reduced by the use of low doses of telmisartan in an Alzheimer’s disease mouse model in vivo [31], [43]. In addition, the epidemiological feasibility of telmisartan was recently identified in a small Alzheimer’s patient cohort [32].

The potential pathways of the PPARγ agonist in Alzheimer’s disease are fairly well known. PPARγ agonists regulate multiple processes, including Aβ homeostasis through the suppression of BACE1 expression, energy metabolism, insulin sensitivity, dyslipidemia, and microglial inflammatory responses [33], [34]. Meanwhile, animal studies suggest that angiotensin receptor blockers decrease Aβ oligomerization [35]. Hajjr et al. provided the first autopsy evidence that angiotensin receptor blockers are associated with reduced amyloid accumulation and Alzheimer’s disease-related pathological change [36]. However, the mechanisms of angiotensin receptor blocker have not been annotated and thus need to be clarified.

**Figure 8:**
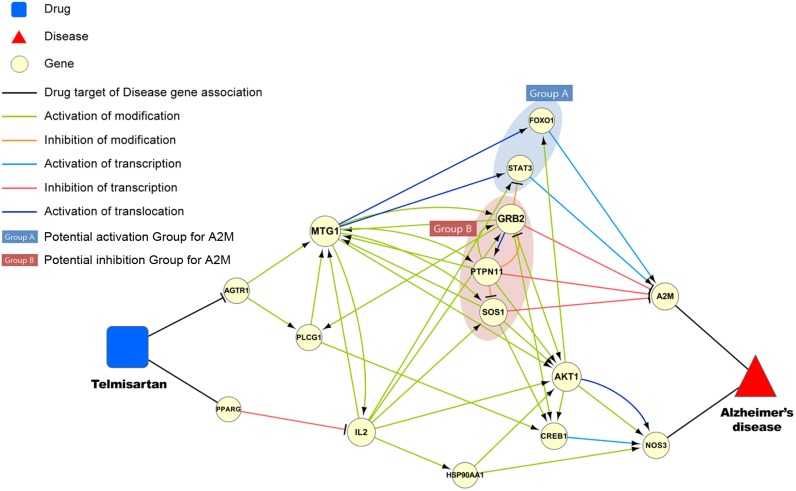
Visualization of potential pathway associated with targets of telmisartan and genes related to Alzheimer’s disease.

We depicted the potential pathway starting from telmisartan to Alzheimer’s disease through a gene regulatory network. In Figure 8, angiotensin II receptor (AGTR1) activates MTG1 to translocate group A genes, which activate the transcription of alpha-2-macroglobulin (A2M). On the other hand, group B genes activated by MTG1 inhibit the transcription of A2M. In other words, the signaling of the angiotensin receptor interrupts the transcription of A2M, which mediates the clearance and degradation of Aβ. The angiotensin II receptor blocker telmisartan may work such that the transcription of A2M is not interrupted. In addition, PPARγ, denoted as PPARG, inhibits the transcription of IL2, which plays a role similar to that of MTG1 in that it partially activates genes in groups A and B.

A GO enrichment analysis for group B shows that the insulin receptor signaling pathway (P = 1.43e-06; FDR = 3.48e-05), the cellular response to an insulin stimulus (P = 3.17e-06; FDR = 6.55e-05) and the response to an insulin stimulus (P = 5.88e-06; FDR = 8.58e-05) are ranked as significantly enriched biological processes. Insulin signaling has a direct role in the development of neurodegenerative diseases [37], and insulin administration improves memory [38]. However, defective insulin signaling is a characteristic feature of the AD brain, and oligomeric amyloid-β induces insulin resistance in the brain [39], [40]. In this regard, Figure 8 shows a potential mechanism of telmisartan, i.e., showing how it blocks the defective insulin signaling cascade, resulting in the inhibition of A2M transcription.

The Aβ peptide mediates synapse loss through cAMP-response element binding protein (CREB) signaling, and the altered CREB signaling plays a crucial role in cognitive dysfunction [41]. In Figure 8, group B, IL2, and their downstream components activate CREB1, resulting in the activation of NOS3 transcription. NOS3 was reported to show various polymorphisms and a significant association with Alzheimer’s disease. Therefore, another potential mechanism of telmisartan may be explained by the inhibition of altered CREB signaling.

Thus, we suggest that the potential mechanism of the angiotensin receptor blocker and the PPARγ agonist is related to defective insulin signaling and altered CREB signaling. Therefore, telmisartan is expected to be a robust candidate drug for Alzheimer’s disease.

## Conclusion

We proposed a method of drug repositioning based on feature extraction from integrative genetic networks and known drug-disease associations. Our method showed high classification accuracy in terms of large-scale prediction of drug indications, and it obtained novel predictions that were validated by their overlap with clinical trials. Furthermore, we discussed interesting examples of novel drug-disease associations at the molecular level. Based on shared pathways of existing drug-disease associations, we can infer more specifically how newly predicted drugs work on the disease. However, there is a limitation in inferring whether a particular drug indication is adverse or effective. For future work, we plan to develop our method for predicting the side effects of existing drugs.

## Supporting Information

Figure S1 **The distribution of Tanimoto scores.**
(TIF)

Table S1 **One sample of actual training data set.**
(XLSX)

Table S2 **The result of ten 10-fold cross-validation runs.**
(XLSX)

Table S3 **Feature contribution.**
(XLSX)

Table S4 **New predictions for 377 drugs and 80 diseases.**
(XLSX)

Table S5 **New predictions for 832 drugs and 239 diseases.**
(XLSX)

Table S6 **Effect of path types toward the prediction accuracy.**
(XLSX)

Table S7 **The AUC reports according to proportion of up- and down-regulated genes.**
(DOCX)

## References

[1] Hopkins AL (2008) Network pharmacology: the next paradigm in drug discovery. Nature chemical biology 4(11): 682–690. 18936753 10.1038/nchembio.118

[2] Cheng F, Liu C, Jiang J, Lu W, Li W, et al (2012) Prediction of drug-target interactions and drug repositioning via network-based inference. PLoS computational biology 8(5): e1002503. 22589709 10.1371/journal.pcbi.1002503 PMC3349722

[3] Chong CR, Sullivan DJ (2007) New uses for old drugs. Nature 448: 645–646. 17687303 10.1038/448645a

[4] Campillos M, Kuhn M, Gavin AC, Jensen LJ, Bork P (2008) Drug target identification using side-effect similarity. Science 321(5886): 263–266. 18621671 10.1126/science.1158140

[5] Schadt EE, Friend SH, Shaywitz DA (2009) A network view of disease and compound screening. Nature reviews Drug discovery 8(4): 286–295. 19337271 10.1038/nrd2826

[6] Keiser MJ, Setola V, Irwin JJ, Laggner C, Abbas AI, et al (2009) Predicting new molecular targets for known drugs. Nature 462(7270): 175–181. 19881490 10.1038/nature08506 PMC2784146

[7] Dudley JT, Deshpande T, Butte AJ (2011) Exploiting drug–disease relationships for computational drug repositioning. Briefings in bioinformatics 12(4): 303–311. 21690101 10.1093/bib/bbr013 PMC3137933

[8] Hu G, Agarwal P (2009) Human disease-drug network based on genomic expression profiles. PLoS One 4(8): e6536. 19657382 10.1371/journal.pone.0006536 PMC2715883

[9] Suthram S, Dudley JT, Chiang AP, Chen R, Hastie TJ, et al (2010) Network-based elucidation of human disease similarities reveals common functional modules enriched for pluripotent drug targets. PLoS computational biology 6(2): e1000662. 20140234 10.1371/journal.pcbi.1000662 PMC2816673

[10] Sirota M, Dudley JT, Kim J, Chiang AP, Morgan AA, et al (2011) Discovery and preclinical validation of drug indications using compendia of public gene expression data. Science translational medicine 3(96): 96ra77–96ra77. 10.1126/scitranslmed.3001318 PMC3502016 21849665

[11] Zhao S, Li S (2012) A co-module approach for elucidating drug–disease associations and revealing their molecular basis. Bioinformatics 28(7): 955–961. 22285830 10.1093/bioinformatics/bts057

[12] Gottlieb A, Stein GY, Ruppin E, Sharan R (2011) PREDICT: a method for inferring novel drug indications with application to personalized medicine. Molecular systems biology 7(1): 496. 21654673 10.1038/msb.2011.26 PMC3159979

[13] Brown KR, Jurisica I (2007) Unequal evolutionary conservation of human protein interactions in interologous networks. Genome biology 8(5): R95. 17535438 10.1186/gb-2007-8-5-r95 PMC1929159

[14] Schaefer CF, Anthony K, Krupa S, Buchoff J, Day M, et al. (2009) PID: the pathway interaction database. Nucleic acids research (suppl 1): D674–D679. 10.1093/nar/gkn653 PMC2686461 18832364

[15] Knox C, Law V, Jewison T, Liu P, Ly S, et al. (2011) DrugBank 3.0: a comprehensive resource for ‘omics’ research on drugs. Nucleic acids research (suppl 1): D1035–D1041. 10.1093/nar/gkq1126 PMC3013709 21059682

[16] McKusick VA (2007) Mendelian Inheritance in Man and its online version, OMIM. American journal of human genetics 80(4): 588. 17357067 10.1086/514346 PMC1852721

[17] Davis AP, Murphy CG, Saraceni-Richards CA, Rosenstein MC, Wiegers TC, et al. (2009) Comparative Toxicogenomics Database: a knowledgebase and discovery tool for chemical–gene–disease networks. Nucleic acids research (suppl 1): D786–D792. 10.1093/nar/gkn580 PMC2686584 18782832

[18] Hall M, Frank E, Holmes G, Pfahringer B, Reutemann P, et al (2009) The WEKA data mining software: an update. ACM SIGKDD explorations newsletter 11(1): 10–18.

[19] Lamb J, Crawford ED, Peck D, Modell JW, Blat IC, et al (2006) The Connectivity Map: using gene-expression signatures to connect small molecules, genes, and disease. science 313(5795): 1929–1935. 17008526 10.1126/science.1132939

[20] Goh KI, Cusick ME, Valle D, Childs B, Vidal M, et al (2007) The human disease network. Proceedings of the National Academy of Sciences 104(21): 8685–8690. 10.1073/pnas.0701361104 PMC1885563 17502601

[21] Wang HM, Liao ZX, Komaki R, Welsh JW, O’Reilly MS, et al (2013) Improved survival outcomes with the incidental use of beta-blockers among patients with non-small-cell lung cancer treated with definitive radiation therapy. Annals of oncology 24(5): 1312–1319. 23300016 10.1093/annonc/mds616 PMC3629895

[22] Barron TI, Connolly RM, Sharp L, Bennett K, Visvanathan K (2011) Beta blockers and breast cancer mortality: a population-based study. Journal of clinical oncology 29(19): 2635–2644. 21632503 10.1200/JCO.2010.33.5422

[23] Melhem-Bertrandt A, Chavez-MacGregor M, Lei X, Brown EN, Lee RT, et al (2011) Beta-blocker use is associated with improved relapse-free survival in patients with triple-negative breast cancer. Journal of clinical oncology 29(19): 2645–2652. 21632501 10.1200/JCO.2010.33.4441 PMC3139371

[24] Powe DG, Voss MJ, Zänker KS, Habashy HO, Green AR, et al (2010) Beta-blocker drug therapy reduces secondary cancer formation in breast cancer and improves cancer specific survival. Oncotarget 1(7): 628. 21317458 10.18632/oncotarget.197 PMC3248123

[25] Al-Wadei HA, Ullah MF, Al-Wadei MH (2012) Intercepting neoplastic progression in lung malignancies via the beta adrenergic (β-AR) pathway: Implications for anti-cancer drug targets. Pharmacological Research 66(1): 33–40. 22487140 10.1016/j.phrs.2012.03.014

[26] Dohlman HG, Thorner J, Caron MG, Lefkowitz RJ (1991) Model systems for the study of seven-transmembrane-segment receptors. Annual review of biochemistry 60(1): 653–688. 10.1146/annurev.bi.60.070191.003253 1652922

[27] Schuller HM (2009) Is cancer triggered by altered signalling of nicotinic acetylcholine receptors? Nature Reviews Cancer 9(3): 195–205. 19194381 10.1038/nrc2590

[28] Cole SW, Sood AK (2012) Molecular pathways: beta-adrenergic signaling in cancer. Clinical cancer research 18(5): 1201–1206. 22186256 10.1158/1078-0432.CCR-11-0641 PMC3294063

[29] Vigil D, Cherfils J, Rossman KL, Der CJ (2010) Ras superfamily GEFs and GAPs: validated and tractable targets for cancer therapy. Nature Reviews Cancer 10(12): 842–857. 21102635 10.1038/nrc2960 PMC3124093

[30] Corbett A, Pickett J, Burns A, Corcoran J, Dunnett SB, et al (2012) Drug repositioning for Alzheimer’s disease. Nature Reviews Drug Discovery 11(11): 833–846. 23123941 10.1038/nrd3869

[31] Tsukuda K, Mogi M, Iwanami J, Min LJ, Sakata A, et al (2009) Cognitive deficit in Amyloid-β–injected mice was improved by pretreatment with a low dose of telmisartan partly because of peroxisome proliferator-activated receptor-γ activation. Hypertension 54(4): 782–787. 19635982 10.1161/HYPERTENSIONAHA.109.136879

[32] Kume K, Hanyu H, Sakurai H, Takada Y, Onuma T, et al (2012) Effects of telmisartan on cognition and regional cerebral blood flow in hypertensive patients with Alzheimer’s disease. Geriatrics & gerontology international 12(2): 207–214. 21929736 10.1111/j.1447-0594.2011.00746.x

[33] Landreth G, Jiang Q, Mandrekar S, Heneka M (2008) PPARγ agonists as therapeutics for the treatment of Alzheimer’s disease. Neurotherapeutics 5(3): 481–489. 18625459 10.1016/j.nurt.2008.05.003 PMC2593876

[34] Kummer MP, Heneka MT (2008) PPARs in Alzheimer’s disease. PPAR research 2008: 8. 10.1155/2008/403896 PMC2465016 18645613

[35] Wang J, Ho L, Chen L, Zhao Z, Zhao W, et al (2007) Valsartan lowers brain β-amyloid protein levels and improves spatial learning in a mouse model of Alzheimer disease. Journal of Clinical Investigation 117(11): 3393–3402. 17965777 10.1172/JCI31547 PMC2040315

[36] Hajjar I, Brown L, Mack WJ, Chui H (2012) Impact of angiotensin receptor blockers on Alzheimer disease neuropathology in a large brain autopsy series. Archives of neurology 69(12): 1632–1638. 22964777 10.1001/archneurol.2012.1010 PMC3608189

[37] Plum L, Schubert M, Brüning JC (2005) The role of insulin receptor signaling in the brain. Trends in Endocrinology & Metabolism 16(2): 59–65. 15734146 10.1016/j.tem.2005.01.008

[38] Craft S, Asthana S, Cook DG, Baker LD, Cherrier M, et al (2003) Insulin dose–response effects on memory and plasma amyloid precursor protein in Alzheimer’s disease: interactions with apolipoprotein E genotype. Psychoneuroendocrinology 28(6): 809–822. 12812866 10.1016/s0306-4530(02)00087-2

[39] Talbot K, Wang HY, Kazi H, Han LY, Bakshi KP, et al (2012) Demonstrated brain insulin resistance in Alzheimer’s disease patients is associated with IGF-1 resistance, IRS-1 dysregulation, and cognitive decline. The Journal of clinical investigation 122(4): 1316. 22476197 10.1172/JCI59903 PMC3314463

[40] Bomfim TR, Forny-Germano L, Sathler LB, Brito-Moreira J, Houzel JC, et al (2012) An anti-diabetes agent protects the mouse brain from defective insulin signaling caused by Alzheimer’s disease–associated Aβ oligomers. The Journal of clinical investigation 122(4): 1339. 22476196 10.1172/JCI57256 PMC3314445

[41] Saura CA, Valero J (2011) The role of CREB signaling in Alzheimer’s disease and other cognitive disorders. Reviews in the neurosciences 22(2): 153–169. 21476939 10.1515/RNS.2011.018

[42] Parkinson H, Kapushesky M, Kolesnikov N, Rustici G, Shojatalab M, et al. (2009) ArrayExpress update–from an archive of functional genomics experiments to the atlas of gene expression. Nucleic acids research (suppl 1): D868–D872. 10.1093/nar/gkn889 PMC2686529 19015125

[43] Shindo T, Takasaki K, Uchida K, Onimura R, Kubota K, et al (2011) Ameliorative effects of telmisartan on the inflammatory response and impaired spatial memory in a rat model of Alzheimer’s disease incorporating additional cerebrovascular disease factors. Biological & pharmaceutical bulletin 35(12): 2141–2147. 10.1248/bpb.b12-00387 23207766

[44] Ahn J, Yoon Y, Park C, Shin E, Park S (2011) Integrative gene network construction for predicting a set of complementary prostate cancer genes. Bioinformatics 27(13): 1846–1853. 21551151 10.1093/bioinformatics/btr283

