# Supplementary figures and images for: A Network-Based Classification Model for Deriving Novel Drug-Disease Associations and Assessing Their Molecular Actions
Source: PLoS One. 2014 Oct 30;9(10):e111668. doi: 10.1371/journal.pone.0111668 (PMC4214731; doi:10.1371/journal.pone.0111668)

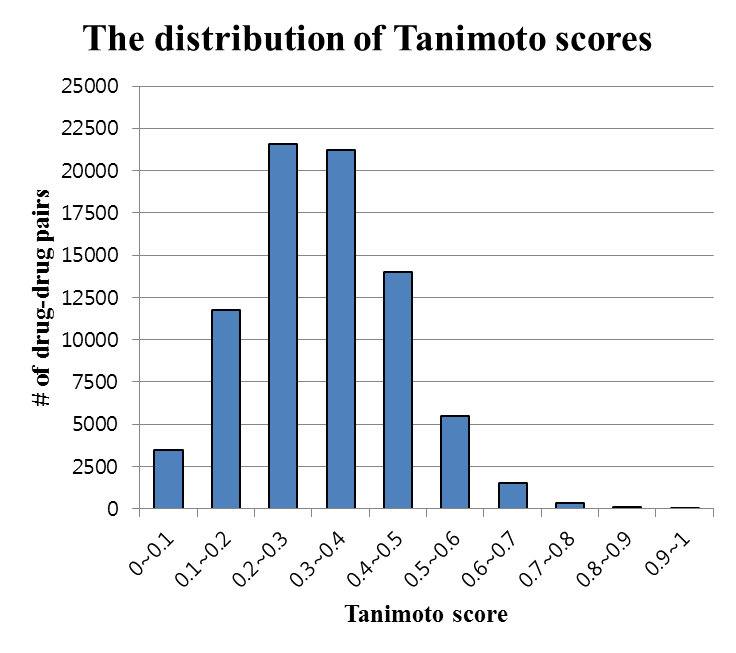

Supplement: Figure S1 — The distribution of Tanimoto scores. (TIF) [file pone.0111668.s001.tif]
