# Supplementary material for: A Network-Based Classification Model for Deriving Novel Drug-Disease Associations and Assessing Their Molecular Actions
Source: PLoS One. 2014 Oct 30;9(10):e111668. doi: 10.1371/journal.pone.0111668 (PMC4214731; doi:10.1371/journal.pone.0111668)
Supplement: Table S7 — The AUC reports according to proportion of up- and down-regulated genes. (DOCX) [file pone.0111668.s008.docx]

**Table S7.** The AUC reports according to proportion of up- and down-regulated genes

| Proportion | 100% | 98% | 96% | 94% | 92% | 90% | 88% | 86% | 84% | 82% | 80% | AVG | STD |
| --- | --- | --- | --- | --- | --- | --- | --- | --- | --- | --- | --- | --- | --- |
| AUC | 0.360 | 0.363 | 0.377 | 0.348 | 0.328 | 0.362 | 0.391 | 0.391 | 0.351 | 0.312 | 0.302 | 0.353 | 0.028 |

AUC: Area Under Receiver Operating Characteristic, AVG: average, STD: standard deviation
